# Correction: An antigenic diversification threshold for falciparum malaria transmission at high endemicity

**DOI:** 10.1371/journal.pcbi.1008945

**Published:** 2021-04-21

**Authors:** The PLOS Computational Biology Staff

There is a textual error in the second-to-last sentence of the abstract. The publisher apologizes for the error. This sentence should read: “This threshold corresponds to an “innovation” number we call *R*_*div*_; it is different from, and complementary to, the one defined by the classic basic reproductive number of infectious diseases, *R*_0_, which does not readily apply under large and dynamic strain diversity.”
